# Distinct immune responses of juvenile and adult oysters (*Crassostrea gigas*) to viral and bacterial infections

**DOI:** 10.1186/s13567-016-0356-7

**Published:** 2016-07-21

**Authors:** Timothy J. Green, Agnes Vergnes, Caroline Montagnani, Julien de Lorgeril

**Affiliations:** IFREMER, IHPE, UMR 5244, Univ. Perpignan Via Domitia, CNRS, Univ. Montpellier, 34095 Montpellier, France; Department of Biological Sciences, Macquarie University, Sydney, NSW 2109 Australia

## Abstract

Since 2008, massive mortality events of Pacific oysters (*Crassostrea gigas*) have been reported worldwide and these disease events are often associated with Ostreid herpesvirus type 1 (OsHV-1). Epidemiological field studies have also reported oyster age and other pathogens of the *Vibrio* genus are contributing factors to this syndrome. We undertook a controlled laboratory experiment to simultaneously investigate survival and immunological response of juvenile and adult *C. gigas* at different time-points post-infection with OsHV-1, *Vibrio tasmaniensis* LGP32 and *V. aestuarianus*. Our data corroborates epidemiological studies that juveniles are more susceptible to OsHV-1, whereas adults are more susceptible to *Vibrio*. We measured the expression of 102 immune-genes by high-throughput RT-qPCR, which revealed oysters have different transcriptional responses to OsHV-1 and *Vibrio*. The transcriptional response in the early stages of OsHV-1 infection involved genes related to apoptosis and the interferon-pathway. Transcriptional response to *Vibrio* infection involved antimicrobial peptides, heat shock proteins and galectins. Interestingly, oysters in the later stages of OsHV-1 infection had a transcriptional response that resembled an antibacterial response, which is suggestive of the oyster’s microbiome causing secondary infections (dysbiosis-driven pathology). This study provides molecular evidence that oysters can mount distinct immune response to viral and bacterial pathogens and these responses differ depending on the age of the host.

**Electronic supplementary material:**

The online version of this article (doi:10.1186/s13567-016-0356-7) contains supplementary material, which is available to authorized users.

## Introduction

The Pacific oyster, *Crassostrea gigas* forms the basis of an important global aquaculture industry with one of the largest annual productions of any marine animals. The Pacific oysters is cultivated on all continents, except Antarctica [1]. Oysters are typically reared in the open environment and are therefore vulnerable to the adverse impacts of disease. Historically, *C. gigas* were chosen in many countries for aquaculture because they were non-native and naturally resistant to many of the protozoan parasites (*Bonamia* spp. and *Marteilia* spp.) that have decimated aquaculture production of indigenous oyster species [2, 3]. Sporadic mortality events of adult *C. gigas* have occurred in many countries over the last four decades [4–6]. These mortality events were often correlated with elevated seawater temperatures and physiological stresses associated with maturation and spawning [reviewed by 5, 6]. No specific pathogen was routinely isolated from these sporadic mortality episodes of *C. gigas* [reviewed by 6]. Surveys often isolated viruses belonging to Ostreid herpesvirus type I (OsHV-1) and bacteria belonging to the *Vibrio* genus [7, 8]. *V. splendidus*-related strains have been isolated from moribund oysters during mortality events [9] and have been widely described regarding their pathogenicity to bivalves. *V. aestuarianus* has been detected in epidemiologic surveys in oysters and the environment since 2001 [8]. OsHV-1 has been detected in mass mortality outbreaks in hatcheries and in epidemiological surveys since 1993 [10].

Since 2008, mass mortality episodes of *C. gigas* have dramatically affected juvenile oysters with high intensities and a wide geographic distribution [7, 11]. This syndrome has been associated with the presence of a particular genotype of Ostreid herpesvirus type 1 (OsHV-1), termed μVar [12]. Mortality associated with this variant was first detected in Europe in 2008 [12], but a closely related genotype has since been associated with mortality of *C. gigas* in New Zealand and Australia in 2010–2011 [13, 14]. In Europe, co-detection of the variant and different *Vibrio* species, including *Vibrio tasmaniensis LGP32* and *V. aestuarianus* have been reported during mortality events of *C. gigas* [7]. These *Vibrio* species are also considered important pathogens in bivalve aquaculture and may be a contributing factor in the mass mortality episodes [8, 15]. Host physiology and ontogeny is another key determinant in the mass mortality episodes of *C. gigas*. Younger age classes of *C. gigas* are more susceptible to OsHV-1 infection [16, 17], whereas older age classes are reported to be more susceptible to *V. aestuarianus* [8]. Thus, determining how host development influences the immunological response and survival of *C. gigas* is a major goal in understanding the recent mass mortality episodes associated with OsHV-1 and different *Vibrio* species.

The immunological response of *C. gigas* against pathogenic viruses and *Vibrio* bacteria are well documented [18–25]. However, most of these studies focused on a single developmental stage and type of pathogen. To date there has not been a controlled laboratory study to simultaneously investigate the effect of oyster age on the immunological response and survival of *C. gigas* infected with three different pathogens. In this study, we address this question by using high-throughput RT-qPCR to measure the immunological response of juvenile and adult *C. gigas* at different time-points post-infection with OsHV-1, *Vibrio tasmaniensis LGP32* and *V. aestuarianus*.

## Materials and methods

### *Crassostrea gigas*

Two different age classes of *Crassostrea gigas* (juvenile and adult) were chosen for experimentation. Juveniles were produced in March, 2012 at the IFREMER oyster hatchery in La Tremblade, Charente-Maritime, France. Juvenile *C. gigas* were on-grown in a biosecure nursery facility before being transferred to IFREMER’s Aquaculture Research Facility in Palavas-les-Flots (Laboratoire Aquaculture en Languedoc, Roussillon, LALR), France. Juveniles were 7 months old at the time of experimentation. Adult *C. gigas* were purchased from an Atlantic oyster farm. These adult oysters were naturally collected in 2009 and grown to maturity in a culture area that doesn’t experience episodes of summer mortality. Adult *C. gigas* were 36 months old at the time of experimentation. Although adults and juveniles used in these experiment do not share the same genetic origins, oysters show very high levels of DNA polymorphism [26] and low genetic differentiation between natural populations in Europe [27] allowing us to compare their transcriptomic patterns. We made sure to use oysters from a multi-parental breeding program to avoid emphasizing any genetic impact on a potential variability of susceptibility on viral and bacterial infections. As mentioned earlier, adult and juvenile animals also share similar life traits of life regarding our trait of interest (i.e. pathogen exposure and defense systems) as they were all grown in hatchery structures and never faced mortality events. Prior to experimentation, juvenile and adult *C. gigas* were tested for OsHV-1 and its variants by qPCR according to [28]. No viral DNA was detected using real time PCR analyses on a sample of 10 individual juvenile and adult animals.

### Pathogens

Ostreid herpesvirus type 1 inoculum was prepared according to Schikorski et al. [29]. The nucleotide sequence of the C region (ORF4) of this OsHV-1 inoculum was PCR amplified according to [30], and had 100% identity to the OsHV-1 variant μVar [31]. This inoculum was estimated to contain 10^8^ OsHV-genomes·µL^−1^ by qPCR [28] and was confirmed to be free of culturable bacteria by plating 50 µL of inoculum on Zobell marine and TCBS agar plates. Two *Vibrio* inoculums were prepared from *V. tasmaniensis* LGP32 and *V. aestuarianus* 02/41 strains. Both strains were isolated from *C. gigas* undergoing a mortality episode [9, 32]. *Vibrios* were grown under agitation at 20 °C in marine broth 2216 (Difco #279110) for 18 h. Cultures of *V.* LGP32 and *V. aestuarianus* were centrifuged (1000 × *g*, 10 min, 20 °C) and resuspended in sterile seawater to an optical density (OD_600_) of 0.50 and 0.05, respectively.

### Experimental challenge

Juvenile (N = 750, Age = 7 months) and adult (N = 750, Age = 36 months) *C. gigas* had a notch filed in their shell adjacent to their adductor muscle using an electric bench grinder. Oysters were then returned to their holding tanks (1 m^3^) that were supplied with continuous renewal of filtered and ultraviolet-sterilised seawater (14 °C) to recover for 24 h. Next, oysters were distributed to 24 aquariums filled with 30 L of seawater (21 ± 1 °C) and allowed to acclimatize for 72 h. Each aquarium had either 50 juveniles or 50 adult oysters. At time 0 h, juvenile and adult oysters were injected in the adductor muscle with an inoculum (see “Pathogen” section) containing either sterile seawater (control), OsHV-1, *V. tasmaniensis* or *V. aestuarianus* (N = 3 replicate aquariums per condition) using a 26-gauge needle attached to a multi-dispensing pipette. Juvenile and adult *C. gigas* were injected with either 50 or 100 µL of inoculum, respectively, according to the size difference of the animals. Following injection, oysters were assessed daily with dead oysters removed from the aquariums and placed in individual plastic bags and snap-frozen with liquid nitrogen and stored at −80 °C for further pathogen detection in moribund oysters. Oysters were defined dead when their shell gaped open and remained sprung open after the oyster was removed from its aquarium.

Three juvenile and three adult *C. gigas* were also sampled from each aquarium at 1, 2, 3, 4 and 7 days post-injection (dpi). The entire oyster was sampled by shucking with a sterile scalpel blade and the three oysters from each aquarium were pooled (three replicate aquariums per condition). Oyster pools were snap-frozen with liquid nitrogen and stored at −80 °C until nucleic acid purification for further gene expression analysis and pathogen detection.

### Nucleic acid extraction and cDNA synthesis

Oyster pools and dead oysters were homogenized by bead-beading (Retsch, Mixer Mill MM400) with a stainless steel ball bearing and housing that had been pre-chilled with liquid nitrogen. Genomic DNA was purified from homogenised oyster tissues using UltraPure Phenol:Chloroform:Isoamyl Alcohol (Invitrogen, #15593-049). Total RNA was purified using TRIzol Reagent (Invitrogen, #15596-018) and DNA contamination eliminated with rDNase I (Ambion, #AM2222). Total RNA and DNA were resuspended to a final concentration of 100 and 20 ng µL^−1^, respectively. First-strand synthesis was performed on 500 ng of total RNA using random hexamer primers (Invitrogen, #48190-011) and M-MLV (Invitrogen, #28025-013). cDNA was diluted 10-fold with sterile water (DNase- and RNase-free) prior to use.

### Pathogen detection and quantification

Detection and quantification of OsHV-1, *V.* LGP32 and *V. aestuarianus* genomic DNA was performed using quantitative PCR (qPCR). All amplification reactions were performed in triplicate using a Roche LightCycler 480 Real-Time thermocycler (qPHD-Montpellier GenomiX platform, Montpellier University). PCR reaction volumes were 6 µL containing LightCycler 480 SYBR Green I Master mix (Roche), 100 nM of pathogen specific primers and 20 ng of DNA. Pathogen specific primer pairs were obtained from the literature [28, 33, 34] and their resulting amplification products were cloned into the pCR4-Topo vector and replicated in *Escherichia coli* DH5a (Invitrogen). Plasmids were extracted using the Wizard Plus SV miniprep DNA purification system (Promega) and standard curves of known concentration of plasmid generated according to the Applied Biosystems manual of absolute real-time RT-PCR quantification [35]. Absolute quantification of OsHV-1, *V.* LGP32 and *V. aestuarianus* genome copies in oyster samples was estimated by comparing the observed Cp values to known plasmid standards. Primer pairs for *V.* LGP32 and *V. aestuarianus* were confirmed not to cross-react.

### High-throughput RT-qPCR and statistical analysis

High-throughput RT-qPCR was performed by the ACOBIOM to assess the transcriptomic response of juvenile and adult *C. gigas* to infection with OsHV-1, *V. aestuarianus* and *V. tasmaniensis LGP32*. We investigated the transcriptional response of 102 immune-related genes, which consisted of 43 putative anti-viral genes and 59 putative anti-bacterial immune genes. It was expected that a proportion of immune genes would be differentially expressed in response to both OsHV-1 and *Vibrio* infection. The antiviral immune genes were identified in the genome and transcriptome of *C. gigas* [36, 37, 51] by performing homology searches (BlastP) using known vertebrate and arthropod antiviral proteins. These proteins have a broad relevance to antiviral immunity, including members of a primitive interferon-response (virus-recognition, signaling), anti-viral effectors, program cell death (autophagy and apoptosis) and RNA interference pathways. Immune genes related to antibacterial immunity were chosen from previous studies investigating the transcriptional response of *C. gigas* to vibriosis [18, 19]. These genes have been identified to be differentially expressed in oysters capable of surviving an experimental infection with a virulent *Vibrio* spp (*V. tasmaniensis* LGP32 and *V. aestuarianus* LPi 02/41) versus an avirulent *Vibrio* sp. (*V. tasmaniensis* LMG20012) [18] or were predictive of the capacity of an oyster to survive a virulent *Vibrio* infection [19]. These 59 immune genes belong to six functional categories, including immunity (recognition, signaling and effector molecules), cellular adhesion and differentiation, cytoskeleton reorganization, apoptosis and oxidative stress. Lastly, three house-keeping genes were also included as internal controls for normalising the data (*Cg*-*EF1,* GenBank #AB122066; *Cg*-*RPL40*, #FP004478; *Cg*-*RPS6*, #HS119070). Additional file 1 provides the GenBank accession number for each target gene, designated immunological function and the nucleotide sequences for each primer pair.

The mRNA expression levels of the chosen target and internal reference genes were determined in juvenile and adult *C. gigas* at 1, 2, 3 and 4 days post-inoculation. The total qPCR reaction volume was 0.5 μL and consisted of 0.25 μL of cDNA (ng) and 0.25 μL of LightCycler^®^ 1536 DNA Green Master Kit (Roche) containing 0.55 μM of PCR primer (Eurogenetec). Pipetting into the 1536 well-plate (Roche) was performed with Labcyte Acoustic Automated Liquid Handling Platform (ECHO). The LightCycler^®^ 1536 Instrument (Roche) was used with the following program: enzyme activation of 95 °C for 1 min followed by 45 cycles of denaturation (95 °C, 2 s) and hybridization-elongation (60 °C, 30 s). A subsequent melting temperature curve of the amplicon was performed to verify the specificity of the RT-qPCR reaction. The amplification efficiency of each primer pair was previously validated using a serial dilution of cDNA and only primer pairs with efficiency of 2 ± 0.1 were used for gene expression analysis. The RT-qPCR data was normalised using the 2^(ΔΔCT)^ method [38] using elongation factor 1 (GenBank AB122066) as the internal reference gene.

Statistical analysis of qPCR data was performed separately for juvenile and adult oysters. Two-way analysis of variance (ANOVA) was conducted to individually assess expression levels of the 102 target genes using the univariate general linear model (GLM) with post hoc Tukey’s HSD test in IBM SPSS Statistics v 20.0. The two factors analysed were “PATHOGEN” with four levels (OsHV-1, *V. tasmaniensis LGP32*, *V. aestuarianus* & seawater) and “TIME” (1, 2, 3 and 4 days). When interactions between these factors were non-significant, these terms were removed from the model to test for single-order effects alone. Hierarchical clustering was performed on target genes identified to be differential expressed using Multiple Array Viewer software (version 4.6.2). Clustering analysis was performed with Pearson correlation based distance.

## Results

### Oyster mortality and pathogen DNA detection

**Figure 1:**
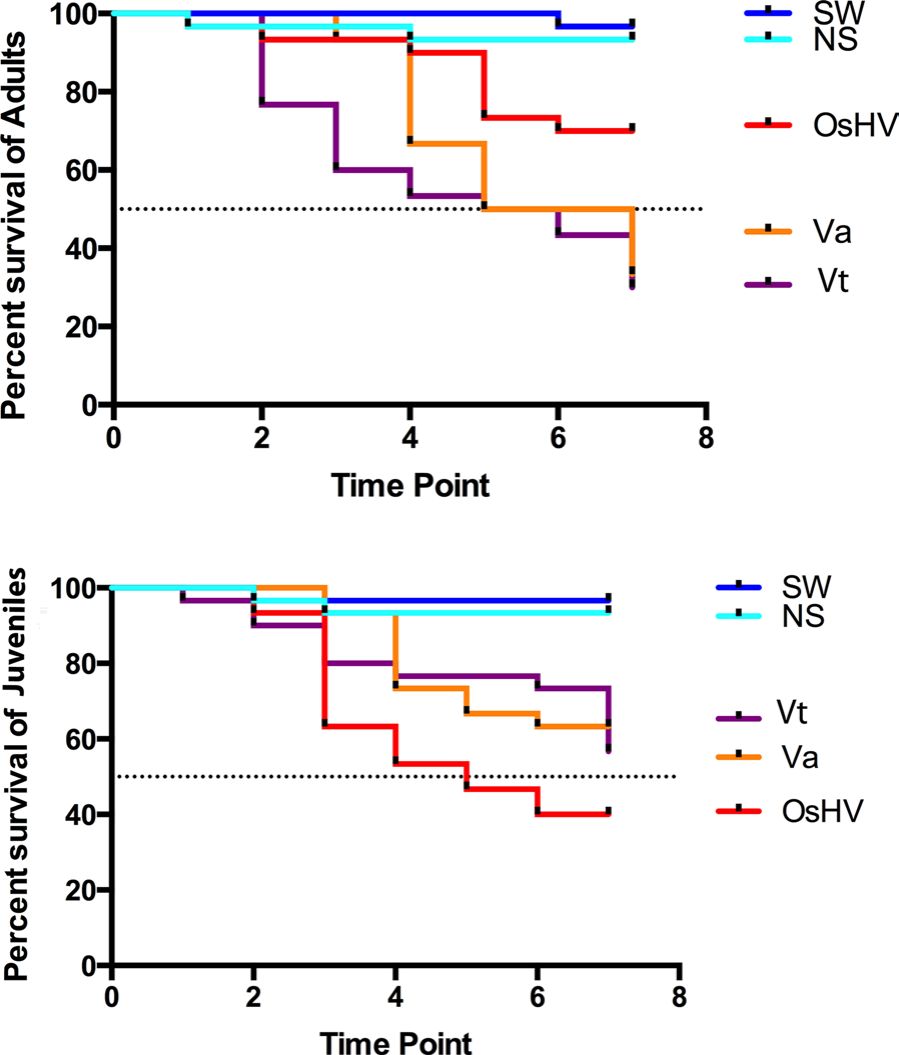
**Kaplan-Meier survival curves.** Survival curves for juvenile (A) and adult (B) oysters injected with seawater (SW, dark blue line) or non-treated (NT, light blue line), OsHV-1 inoculum (red), *Vibrio tasmaniensis* LGP32 (yellow), *V. aestuarianus* (purple).

**Figure 2:**
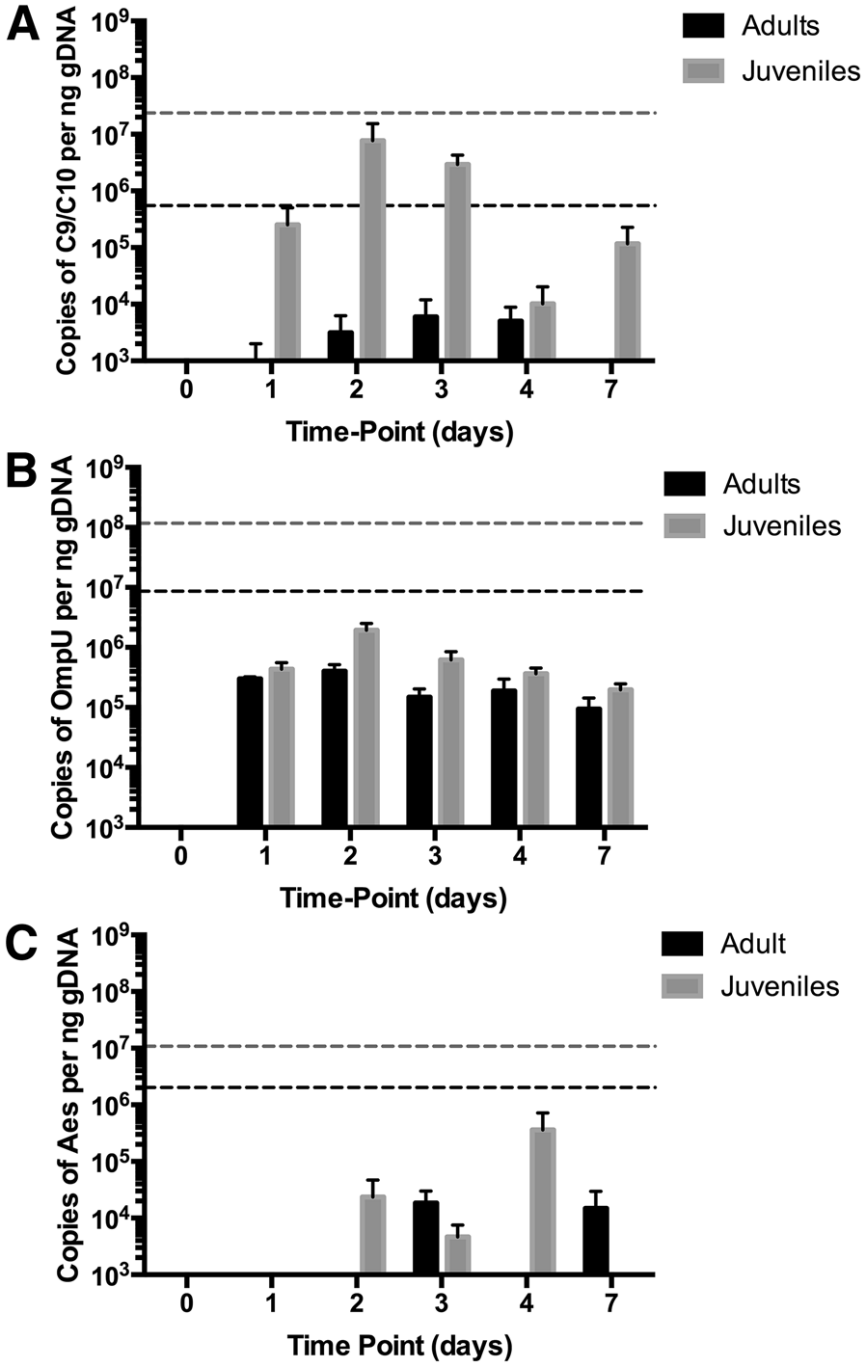
**Pathogen load in juvenile and adult oysters.** The concentration of OsHV-1 (Graph** A**), *Vibrio* LGP32 (Graph** B**) and *Vibrio aestuarianus* (Graph** C**) DNA is shown in the tissues of adult (black bars) and juvenile (grey bars) *Crassostrea gigas* (mean ± SD). The concentration of pathogen DNA was below detectable limits at 0 h post injection. Horizontal lines represent the average concentration of pathogen DNA in the tissues of moribund adult (dash black line) and juvenile (dash grey line) *C. gigas.*

Minimal mortality of controls *Crassostrea gigas* occurred during experimentation (juvenile and adult >97% survival, sterile seawater injection or non-treated controls). Juveniles were more susceptible to OsHV-1 infection than adult *C. gigas* (Figure 1). Overall, 60% of juveniles succumbed to OsHV-1 with peak mortality occurring 3 dpi leading to 40% survival 7 dpi (Figure 1A) whereas, 30% of adult *C. gigas* succumbed to OsHV-1 and mortality peaked on day 5 leading to 70% survival 7 dpi (Figure 1B). The concentration of OsHV-1 DNA in juvenile and adult tissues was below detectable limits on day zero, but rapidly increased following injection with the OsHV-1 inoculum. The average concentration of OsHV-1 DNA in juveniles peaked at 2 dpi with 7.7·10^6^ genome copies ng^−1^ of genomic DNA (Figure 2A). The concentration of OsHV-1 DNA in adult oysters was consistently lower than in juveniles and peaked at 3–4 dpi with 4·10^3^ genome copies ng^−1^ of genomic DNA (Figure 2A). The average viral DNA in moribund juvenile and adult tissues was considerably higher at 2.4·10^7^ and 5.5·10^5^ genome copies ng^−1^ of genomic DNA, respectively.

In contrast to OsHV-1 infection, adult *C. gigas* were more susceptible to *Vibrio* infection than juveniles (Figure 1). Overall, 63 and 70% of adult *C. gigas* died of *V. aestuarianus* and *V. tasmaniensis* LGP32 infection, respectively (37 and 30% survival, Figure 1A). *V. tasmaniensis* LGP32 infection induced mortality of adult *C. gigas* was rapid with peak mortality occurring at 2 dpi, whereas peak mortality of adult *C. gigas* infected with *V. aestuarianus* occurred later at 4 dpi (Figure 1B). Juveniles suffered 37 and 43% mortality to *V. aestuarianus* and *V. tasmaniensis* LGP32, respectively (63 and 57% survival—Figure 1B). The average concentration of *V. tasmaniensis* LGP32 DNA peaked at 2 dpi, at 2·10^6^ and 4·10^5^ genome copies.ng^−1^ of DNA isolated from juvenile and adult tissues (Figure 2B). Whereas, the average concentration of *V. aestuarianus* DNA peaked later at 4 and 3 dpi for juvenile and adult tissues, respectively (Figure 2C). The average concentration of *V. tasmaniensis* LGP32 DNA in moribund oysters reached 10^8^ and 10^7^ genome copies of *V. tasmaniensis* LGP32 ng^−1^ of genomic DNA isolated from individual juvenile and adult moribund oysters, respectively. The average concentration of *V. aestuarianus* DNA was higher in moribund oysters with 10^7^ and 2·10^6^ genome copies of *V. aestuarianus*.ng^−1^ of genomic DNA isolated from individual juvenile and adult moribund oysters, respectively.

### Distinct immune response in juvenile and adult *C. gigas*

The temporal expression profile of 102 target genes and 3 internal reference genes was analysed by high-throughput RT-qPCR to determine the transcriptional response of juvenile and adult *C. gigas* to pathogen infection at 1, 2, 3 and 4 days post-infection. The high-throughput RT-qPCR analyses generated 10 080 individual Cq values. Statistical analysis revealed that a total of 30 and 27 target genes were differentially expressed in response to pathogen infection in juvenile and adult *C. gigas*, respectively (two-way ANOVA, *p* < 0.05) (see Additional file 2 for details). The majority of genes regulated in response to pathogen infection in juvenile and adult *C. gigas* belonged to the following functional categories: cellular adhesion (i.e. neural-cadherin), heat-shock proteins (i.e. sHSP, HSP 68 kDa), apoptosis (i.e. TNF ligand and IAP), non-self recognition (i.e. galectin) and pathogen recognition (i.e. toll-like receptors, rig-like receptors, c-type lectins), signaling molecules (i.e. MyD88, IκB, IRF-8 and SOC-1), antiviral effectors (i.e. viperin, ADAR-L, OAS, PKR and IFI44) and antimicrobial peptides (i.e. big defensin).

**Figure 3:**
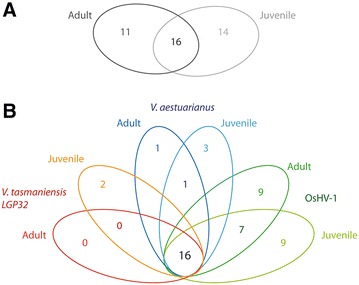
**Venn diagram of differentially expressed genes in juveniles and adults under the challenge of OsHV-1,** ***V. aestuarianus*** **and** ***V. tasmaniensis LGP32***.** A** Comparison between juveniles and adults.** B** Comparison between challenges.

Comparison of transcriptome data from juvenile and adult *C. gigas* revealed specific response to juvenile or adult stages. Sixteen genes were common to the early response to pathogen in juveniles and adults with 14 and 11 specific to the adult or juvenile response, respectively (Figure 3A). Several immune genes are also differentially expressed in adults and juveniles in response to the same pathogen. Concerning specific response of juveniles compared to adults, 9 genes were found to be specifically regulated in response to OsHV-1, 3 to *V. aestuarianus* and 2 to *V. tasmaniensis LGP32* (Figure 3B). In the case of OsHV-1 infections, inhibitor of apoptosis (IAP) are up-regulated in juvenile (*p* < 0.05), but OsHV-1 μVar infection does not influence the expression of IAP in adults (*p* > 0.05). Signaling molecules (IRAK-4, IκB) in the NF-κB pathway are also exclusively up-regulated in juveniles inoculated with OsHV-1 μVar (*p* < 0.05), but not in adults. For *Vibrio* infections, several genes also appeared differentially expressed specifically at one developmental stage. Some genes appear only regulated in juveniles as big defensin, TGF, iIntegrin-binding protein, and cdc42 homolog. Concerning specific response of adults compared to juveniles, 10 genes were found to be specifically regulated in response to OsHV-1, and 1 to *V. aestuarianus* (Figure 3B). Interestingly, only adult *C. gigas* differentially express the autophagy related protein (Atg8/LC3) in response to pathogen infection (*p* < 0.05). Other genes differentially expressed in adult *C. giga*s include gamma interferon inducible lysosomal thiol reductase (GILT-1, GILT-2), interleukin receptor, a c-type lectin, early growth response protein (EGR), poly(U) endoribonuclease, MEGF10, metallothionein and extracellular superoxide dismutase (EcSOD) (*p* < 0.05).

### Distinct early response to viral and bacterial infection

**Figure 4:**
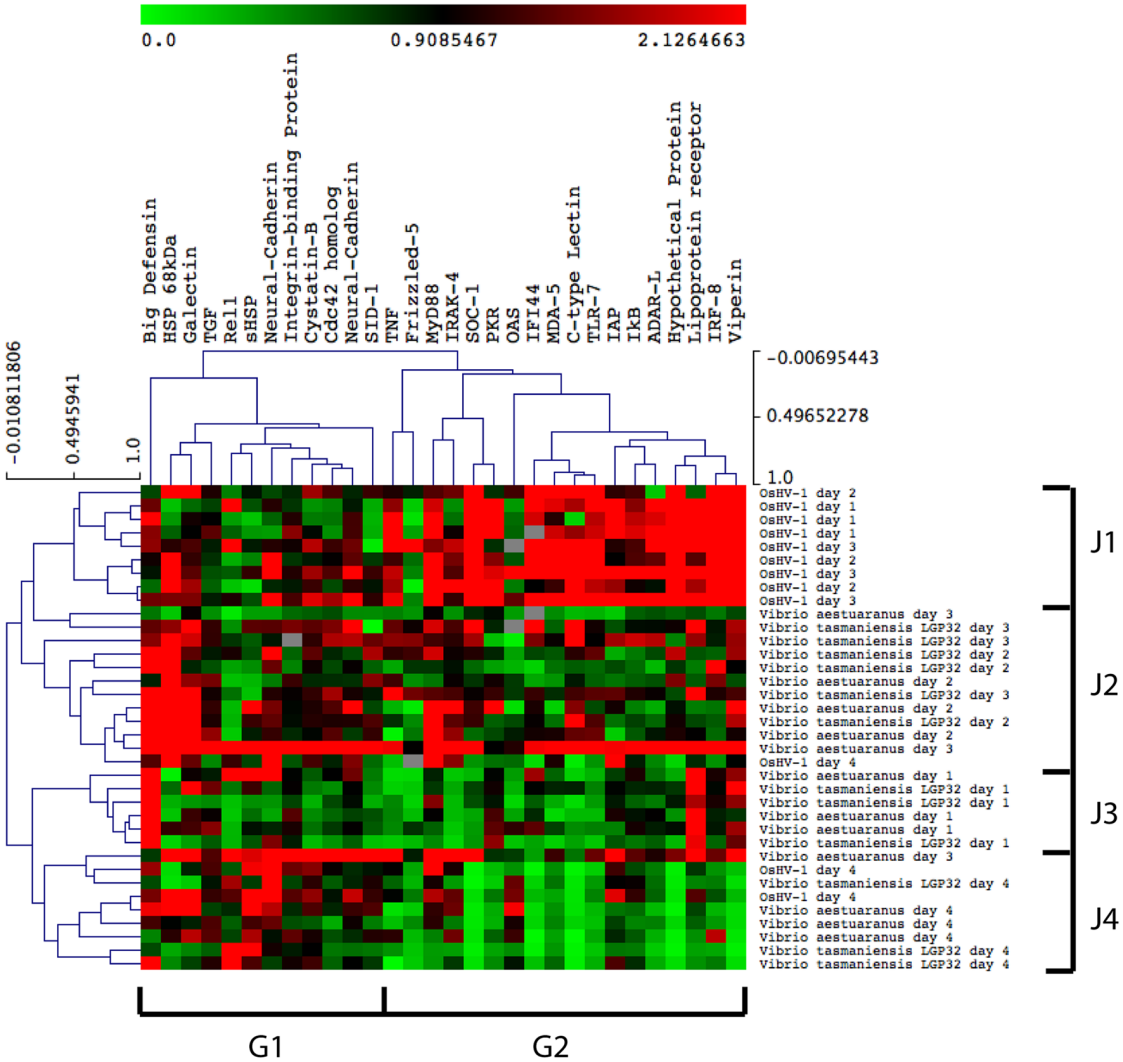
**Heat map depicting the expression of immune genes of juvenile** ***Crassostrea gigas*** **infected with OsHV-1,** ***Vibrio aestuarianus*** **and** ***V. tasmaniensis*** **LGP32.** The expression of each target gene was normalised to the transcript level of control groups (juvenile injected with sterile seawater) at each sampling time point (1, 2, 3 and 4 days post-injection). Hierarchical clustering confirms that juvenile have a distinct anti-viral and anti-bacterial response. Juveniles in the early stages of OsHV-1 infection (J1) cluster separately to juveniles in the early (J3), mid (J2) and late (J4) stages of *Vibrio* infection. No distinction in the transcriptional response was observed between juvenile infected with *V. aestuarianus* or *V.LGP32*. Juveniles in the early stages of OsHV-1 infection have a transcriptional response involving the up-regulation of genes in cluster G2, whereas *Vibrio* infection results in the up-regulation of genes in cluster G1.

**Figure 5:**
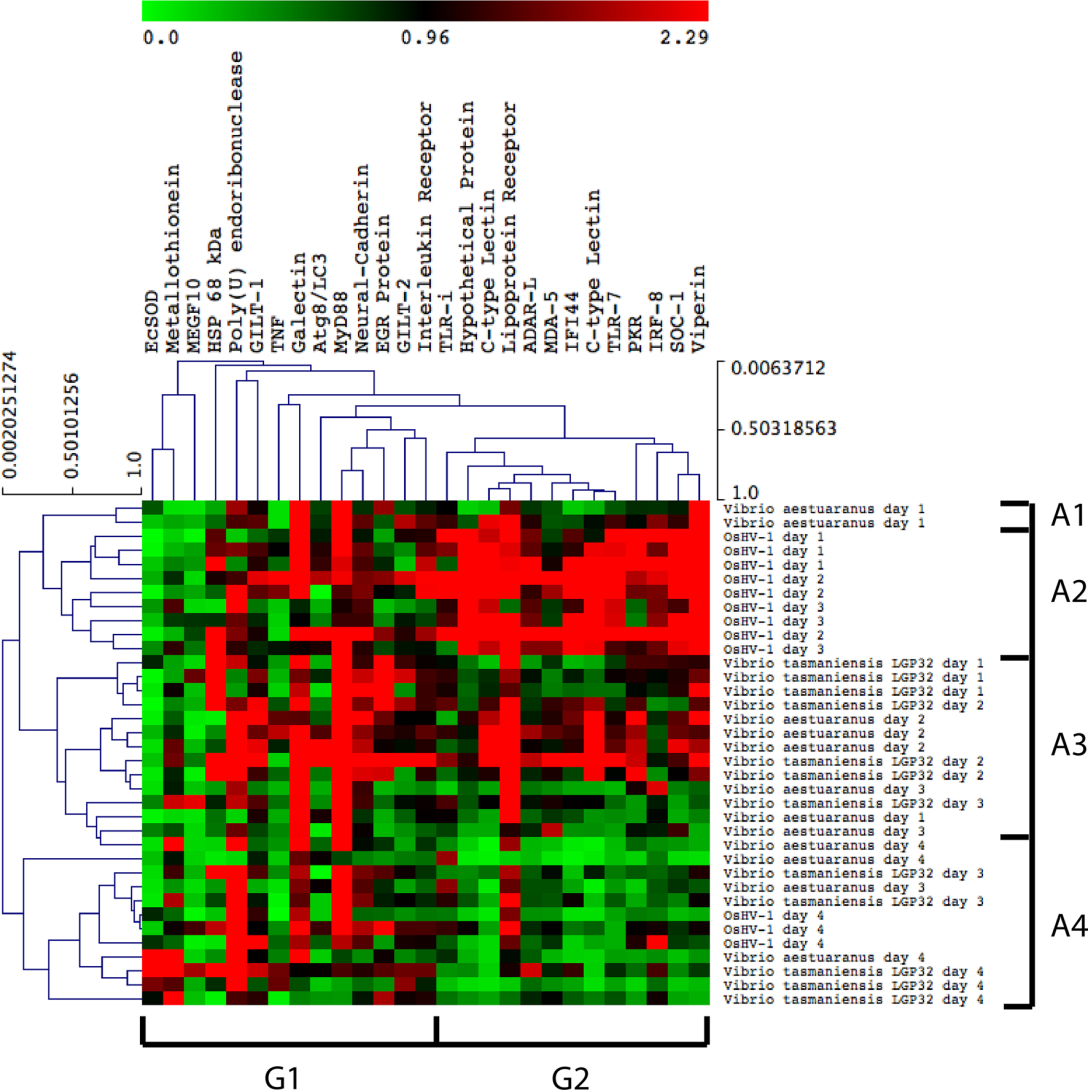
**Heat map depicting the expression of immune genes of adult** ***Crassostrea gigas*** **infected with OsHV-1,** ***Vibrio aestuarianus*** **and** ***V. tasmaniensis*** **LGP32.** The expression of each target gene was normalised to the transcript level of control groups (adults injected with sterile seawater) at each sampling time point (1, 2, 3 and 4 days post-injection). Adult oysters in the early stages of OsHV-1 infection (A2) cluster separately to adult oysters in the early (A3) and late (A4) stages of *Vibrio* infection. Hierarchical clustering did not reveal a distinction in the transcriptional response of adult *C. gigas* infected with *V. aestuarianus* or *V. tasmaniensis* LGP32.

Hierarchical clustering of these differentially expressed genes in response to pathogen infection across all time points revealed juvenile and adult *C. gigas* have a distinct molecular signature to viral and bacterial infection (Figures 4 and 5). Adult and juvenile transcriptomic data were treated separately according to their differences in life stages and backgrounds. Figure 4 revealed the transcriptional response of juveniles in the early stages of OsHV-1 infection (juvenile cluster J1) involves the regulation of a group of immune genes involved in apoptosis and a primitive interferon response (gene cluster G2). In contrast, juveniles in the early (cluster J3), mid (cluster J2) and late (cluster J4) stages of *Vibrio* infection have a distinct transcription response involving regulation of a set of genes involved in a broad range of functions, including cellular proliferation (i.e. TGF ligand), adhesion (i.e. neural-cadherin and integrin-binding protein), migration (i.e. CDC42 homolog), and immune response (i.e. galectin, big defensin, HSPs and cystatin B) (gene cluster G1). Interestingly, genes from the G2 cluster globally appeared not regulated in response to bacterial infection. There was no clear distinction in the transcriptional response of spat to *V. aestuarianus* and *V. tasmaniensis* LGP32 (Figure 4).

As for juveniles, adult *C. gigas* displayed a distinct transcriptional response to OsHV-1 μVar and *Vibrio* infection (Figure 5). Hierarchical clustering reveals adult oysters in the early stages of OsHV-1 infection (adult cluster A2) also have a distinct anti-viral response involving up-regulation of interferon-related genes (gene cluster G2). Whereas, adult *C. gigas* infected with *Vibrio* cluster into an early (clusters A1 and A3) and late (cluster A4) groups (Figure 5) involving differential gene expression of a set of genes involved in molecular functions, including cellular differentiation (i.e. poly(U)-specific endoribonuclease-D and TNF), cellular adhesion (i.e. neural-cadherin), immune response (i.e. HSP 68 kDa, galectin, interleukin receptor, MyD88), and oxidative stress (i.e. EcSOD) (gene cluster G1). Similar to juveniles, no clear distinction was observed in the transcriptional response of adult *C. gigas* to *V. aestuarianus* and *V. tasmaniensis* LGP32 (Figure 5).

The transcriptional response of juvenile and adult *C. gigas* in the later stages of OsHV-1 infection (4 dpi) is similar to *C. gigas* samples inoculated with *Vibrio* (Figures 4 and 5).

## Discussion

This study provides the first direct comparison of the transcriptional responses of juvenile and adult *Crassostrea gigas* to viral (OsHV-1) and bacterial (*Vibrio tasmaniensis* LGP32 and *V. aestuarianus*) pathogens. Data from this experiment provides some important insights into the molecular basis of the mass mortality events of cultivated *C. gigas* that are currently occurring in many countries [12–14, 17]. The observed mortality in our experiment corroborates recent epidemiological field studies that revealed juveniles are more susceptible to viral infection with OsHV-1 [16, 17] and reports that adult *C. gigas* are more susceptible to bacterial infection with *Vibrio* [39]. Although juvenile oysters have been demonstrated to also be susceptible to *V. aestuarianus* in controlled experiments, this pathogen has been predominantly associated with adult oyster mortality events and adult oysters seem to be more susceptible to this bacterium than spat (i.e. young juvenile oysters) [8, 39]. We measured the concentration of OsHV-1 DNA in juvenile and adult tissues by qPCR (Figure 2A), which revealed the rate of OsHV-1 replication is greater in juveniles with the maximum concentration of OsHV-1 DNA occurring earlier in juveniles (Figure 2A). The earlier peak in OsHV-1 DNA coincides with mortality occurring earlier for juveniles compared to adults (Figure 1). Our results are in accordance with previous studies where the level of cumulative mortality using injection as the method of OsHV-1 inoculation varies according to the age and genetics of the host, but typically ranges between 15 and 90% [21, 22, 25, 29, 40, 41]. However, if OsHV-1 is known to be harmful to larvae, spats and juveniles, it has been generally accepted that adults are much more resistant to the virus and found to be asymptomatic carriers. Consequently, little attention has been paid to adult susceptibility to OsHV-1. A few studies have recorded vial DNA loads in adult oysters, which were associated with lower mortalities and no clear link was established with abnormal mortalities [16, 17, 42–47]. It has been suggested that these observations could reflect the fact that adult oysters may have survived previous mortality events and consequently became resistant to the disease [17, 42, 43, 48]. However, more recent studies, concurring with our results, have confirmed that juveniles mortalities are more important, but also that adult oysters can be susceptible to viral infection in experimental conditions with mortalities reaching a mean of 27.5% at 6 days post-infection [21]. The authors suggested that in the case of adults, the apoptosis pathway might be involved to circumvent virus infection, ultimately reducing the viral DNA load in oyster tissues along the time of experiment [21].

Transcriptome data from high-throughput RT-qPCR analysis of the *C. gigas* immune response also provides useful insights into the molecular basis for the massive mortality events. Hierarchical clustering analysis of differentially expressed genes revealed the transcriptomic response of juvenile and adult *C. gigas* inoculated with *V. tasmaniensis* LGP32 and *V. aestuarianus* was distinctly different to *C. gigas* in the early stages of OsHV-1 infection (Figures 3 and 4). These observations suggest the immune response of *C. gigas* can distinguish and tailor specific responses to bacterial and viral pathogens.

In the early stages of OsHV-1 infection, juvenile and adult *C. gigas* displayed a specific antiviral response involving genes related to apoptosis (TNF ligand, IAP), virus recognition (i.e. TLR, MDA-5, C-type lectins, frizzled, lipoprotein receptor), immune-signaling (i.e. MyD88, IκB, IRAK, IRF-8, SOC-1) and antiviral effectors (i.e. viperin, IFI44, ADAR-L, PKR). Some studies have suggested that the higher mortality of juveniles is because their immune system is immature [13], but our results, concurring with recent studies [21–23, 25], clearly showed that juveniles, as for adults, are able to mount an antiviral response, which is manifested by the regulation of these numerous immune genes. Moreover, we showed here those juvenile oysters are more able to circumvent bacterial infection as opposed to adults. Interestingly, adults and juveniles seem to demonstrate a common response to OsHV-1 through an IFN-like pathway. Animals in the vertebrate phylum express the same recognition, signaling and effector genes in response to viruses, dsRNA and interferon cytokines [49, 50], which has led several authors to conclude that *C. gigas* have a interferon-like response [25, 51, 52]. The antiviral effectors induced by interferon control viruses by directly targeting pathways and functions required during the viruses life-cycle, including cellular entry, translation and replication of the viruses genome, and exit in order to infect new cells [50]. All of the effector molecules (viperin, ADAR-L, IFI44, OAS and PKR) induced by OsHV-1 are known to have direct antiviral activity by inhibiting virus replication via targeting synthesis (transcription and translation) of both host and viral proteins in vertebrates [reviewed by 50]. The number and magnitude of antiviral effectors induced by juvenile *C. gigas* was greater than adults, which may result from the higher concentration of OsHV-1 DNA in juvenile tissues (Figure 2). Previous studies indeed show a positive correlation between the expression level of specific immune genes (MyD88, IFI44, IκB2) and viral DNA loads [22]. From the host perspective, having a weaker antiviral response may be preferable to over-expressing a group of extremely potent antiviral effectors, as the latter could result in a toxic cellular environment [53]. In addition, we noticed that juveniles, in contrast to adults, are specifically regulating a number of NF-κB pathway components (IRAK, IκB, MyD88) and a distinct TLR. These genes were also up-regulated in previous studies, confirming our results [21–23, 25]. Although there is a functional link between the non-canonical NF-kB and the IFN response [54, 55], the NF-κB pathway is commonly regarded as a major regulator of the innate immune defense to bacterial or viral infection [56, 57]. This pathway is activated in response to a variety of stimuli, including viral and bacterial infections, exposure to pro-inflammatory cytokines, mitogens and growth factors, and stress-inducing agents [57–59]. Moreover, several studies indicate that viruses have acquired the capability to reprogram NF-κB antiviral activity and to exploit the factor for efficient replication [60]. These finding might reveal that juveniles are unable to properly tailor their antiviral response and display a more pleiotropic response resulting in different susceptibilities or response efficiencies.

Hierarchical clustering analysis failed to differentiate a clear transcriptional response between *V. tasmaniensis* LGP32 and *V. aestuarianus* (Figures 4 and 5), which suggests either that the immune response of *C. gigas* does not distinguish between these bacterial pathogens. Although, Zhang et al. showed that the transcriptional response to distinct bacteria and vibrios can vary, to our knowledge, there is no record of comparison between these vibrios strains [23]. Further global transcriptomic analyses will be needed to access the subtle difference that may exist between these two responses. However, temporal changes in the transcriptional response to *Vibrio* infection were evident with expression profiles clustering into early and late responses (Figures 3 and 4). Interestingly, *C. gigas* in the latter stages of OsHV-1 infection had a transcriptomic response that is undistinguishable to *C. gigas* infected with *Vibrio*. Bacteria belonging to the *Vibrio* genus are known to be a common component of the oyster’s microbiome [61, 62] and the transcriptomic data might suggest *Vibrio* were causing a co-infection in the later stages of the OsHV-1 challenge. Indeed, the detection frequency and quantity of *Vibrio* DNA in *C. gigas* tissue is known to increase markedly during field mortality events where OsHV-1 is diagnosed as the primary pathogen [42, 63]. Our transcriptomic data provides further support of a multi-pathogen etiology in the massive mortality events currently occurring in Europe [15, 42].

In summary, we showed that oysters are able to mount distinct immune responses to bacterial or viral pathogens. These responses differ depending on the age of the animals. These data provide pathogen specific sets of genes and a unique opportunity to further investigate the role played by different pathogens in these multifactorial mortality events. Future research should focus on investigating the role of the oyster’s microbiome in the development of the mass mortality events of *C. gigas* overwhelming the majority of countries that farm the Pacific oyster. Such studies would help to decipher the role played by other opportunistic pathogens during the course of the mass mortality events that are associated with Ostreid herpesvirus type 1 infection.

## Additional files

10.1186/s13567-016-0356-7 **List of primer from antiviral and antibacterial gene selection.** Table listing primers used in the high throughput RT-qPCR analysis with their respective nucleic sequence, Genbank reference and BLAST hits.
10.1186/s13567-016-0356-7 **List of regulated genes in juveniles and adults in response response to OsHV-1,** ***V. aestuarianus*** **and V** ***. tasmaniensis LGP32***. Statistical analysis of qPCR data was performed separately for juvenile and adult oysters. Two-way analysis of variance (ANOVA) was conducted to individually assess expression levels of the 102 target genes using the univariate general linear model (GLM) with post hoc Tukey’s HSD test in IBM SPSS Statistics v 20.0.
